# Digital literacy as a shield: A systematic review of strategies, pitfalls, and outcomes in preventing digital dangers among children

**DOI:** 10.1192/j.eurpsy.2026.11352

**Published:** 2026-07-31

**Authors:** M. Sharifi, C. A. Lewis, M. Firoozi, S. Khani, P. Afkari

**Affiliations:** 1 Psychology, Leeds Trinity University, Leeds, United Kingdom; 2 Psychology, Islamic Azad University, Bandargaz Branch, Bandargaz; 3 Psychology; 4 Psychology and Educational Sciences, University of Tehran; 5 Psychology, Payam-e-noor University, Tehran, Iran, Islamic Republic Of

## Abstract

**Introduction:**

Objective: Children’s growing digital engagement exposes them to risks such as cyberbullying, privacy breaches, and harmful content. Digital literacy, the ability to critically and responsibly navigate technology, has emerged as a key protective strategy. This review synthesizes evidence on the effectiveness and challenges of digital literacy interventions for children.

**Objectives:**

This review synthesizes evidence on the effectiveness and challenges of digital literacy interventions for children.

**Methods:**

Following PRISMA guidelines, we systematically searched PubMed, Scopus, Web of Science, ERIC, and PsycINFO for studies published between 2012 and 2024. Eligible studies evaluated digital literacy interventions among children aged 6–14 years-old, reporting outcomes on online risk reduction and safer digital behaviors. Data were synthesized narratively and, where possible, meta-analytically.

**Results:**

Thirty-four studies met inclusion criteria, including randomized trials, quasi-experimental designs, and qualitative research. Most interventions were delivered in schools or through interactive online platforms. Findings showed that strengthened digital literacy skills consistently reduced cyber bullying incidents, privacy violations, and exposure to harmful content. Effective strategies involved age-appropriate, experiential learning, parental and teacher involvement, and gamified or scenario-based content. Common pitfalls included limited teacher training, lack of cultural adaptation, and inequities in access and participation. Sustained improvements were most evident in programs with longitudinal reinforcement and ongoing support.

**Conclusions:**

Digital literacy is an evidence-based approach to safeguarding children online; however, its effectiveness depends on context-sensitive strategies and overcoming barriers such as unequal access and educator preparedness. Future work should prioritize scalable, adaptive interventions and cross-sector partnerships to close the digital safety gap.

**Disclosure of Interest:**

None Declared

